# A Bilateral Traumatic Hip Obturator Dislocation

**DOI:** 10.1155/2016/3145343

**Published:** 2016-02-08

**Authors:** Ahmet Adnan Karaarslan, Nihat Acar, Tolga Karci, Erhan Sesli

**Affiliations:** ^1^Orthopedics and Traumatology Department, Şifa University Faculty of Medicine, Sanayi Street, No. 7, Bornova, 35190 Izmir, Turkey; ^2^Ozel Gazikent Medical Center Department of Orthopedics and Traumatology, Gaziemir, 35410 Izmir, Turkey

## Abstract

A case of a bilateral simultaneous traumatic obturator dislocation of both hip joints in an 18-year-old young man following a traffic accident is presented. We reduced the dislocated femoral heads immediately under general anesthesia followed by passive and active exercises and early full-weight bearing mobilization. After 5 years, the result was excellent.

## 1. Introduction 

Traumatic anterior hip joint dislocations are less frequent than posterior ones. Bilateral traumatic hip dislocations are extremely rare [1, 2]. We report the functional result of a bilateral traumatic obturator hip dislocation treated with immediate reduction.

## 2. Case Report

**Figure 1:**
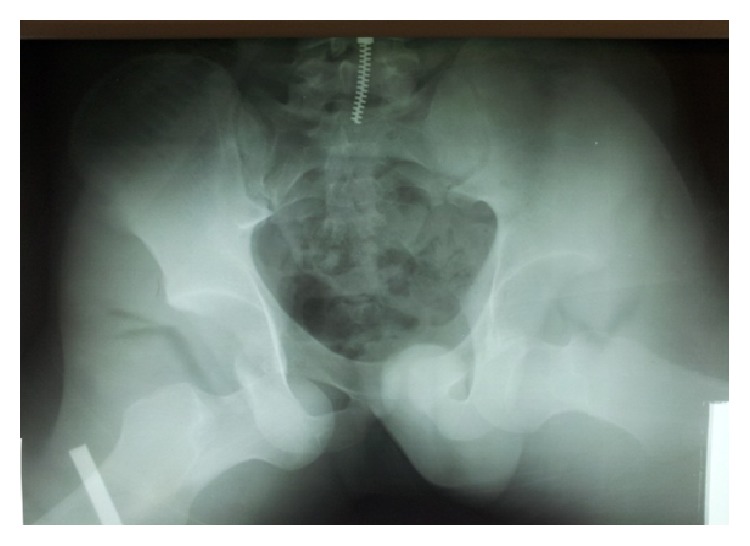
X-ray of a bilateral simultaneous traumatic obturator dislocation of both hip joints.

**Figure 2:**
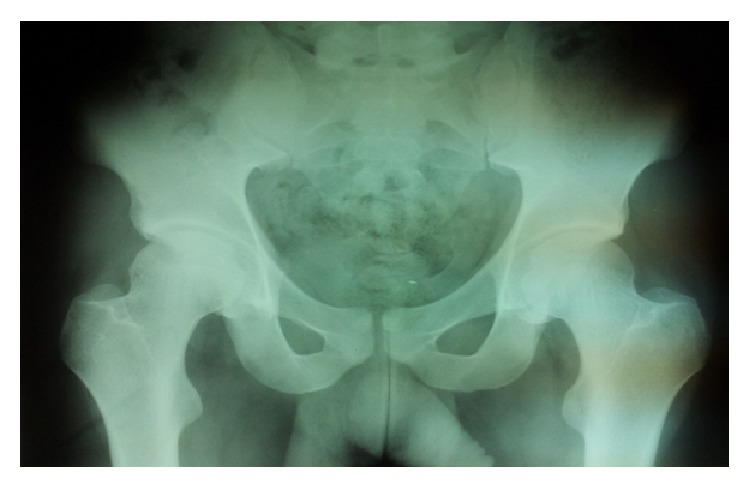
Concentric reduction of both femoral heads after close reduction.

An 18-year-old man sustained a severe bilateral hip pain after a motorcycle accident. At the time of accident the patient was driving his motorcycle with his both hips in external rotation, abduction, and flexion. The accident happened while the patient was riding the motorcycle with a high speed crushing a vehicle hardly. When he was subjected to a sudden deceleration, both hips went to forced external rotation, abduction, and flexion, resulting in bilateral obturator dislocation. He was referred to the emergency department with a bilateral lower extremity abduction, flexion, and external rotation. Physical examination of both lower extremities revealed a 70° abduction, a 70° external rotation, and 60° flexion of both hips. Both femoral heads were palpable in the right and left obturator region. Passive and active motion of the hips were impossible. No neurovascular impairment was detected and X-ray revealed a bilateral traumatic obturator dislocation of both hip joints (Figure 1). 30 minutes after the accident the patient was brought to the operating theater and, under general anesthesia, closed reduction of both hip joints was carried out with longitudinal traction of lower extremities using Allis's maneuver. After close reduction of both hip joints, stability and range of motion were examined; X-ray showed a concentric reduction of both femoral heads (Figure 2). There was no associated injury or fractures on MRI. Physiotherapy was initiated at the first week with quadriceps and gluteal muscles isometric exercises and passive hip movements were performed. At the second week active-assisted range of motion was done. After the sixth week, the patient was mobilized with partial weight bearing using a pair of axillary crutches. At the second month the patient was mobilized with full-weight bearing. The patient returned to his full activities at three months. After five years the patient was pain-free with a full range of motion of his both hips and there were no MRI signs of avascular necrosis of the femoral heads.

## 3. Discussion 

Bilateral simultaneous traumatic obturator dislocations are extremely rare. The greater trochanter impinges on the acetabular rim (on the ileum) which in turn works as a lever arm, extracting the femoral head out of the acetabulum. The degree of hip flexion usually determines the type of anterior dislocation (superior or inferior). It has been reported that the obturator dislocation results from simultaneous external rotation, abduction, and flexion of the hip joint [3]. Traffic accidents are considered the main cause of bilateral anterior hip dislocations in the majority of cases with dashboard impact [2].

At the time of accident the patient was sitting on his motorcycle with his both hips in external rotation, abduction, and flexion. When he was subjected to a sudden deceleration, both hips went to forced external rotation, abduction, and flexion. This position of both hip joints resulted in a bilateral obturator dislocation. In anterior hip dislocations, the neurovascular status of the lower limb should be evaluated and the patient should be examined carefully for any concomitant injuries [4, 5].

Prompt reduction should be made immediately under general anesthesia to prevent complications because bilateral obturator dislocation is considered as a true emergency. Avascular necrosis, traumatic arthritis, and myositis ossificans are late complications of hip dislocations. The incidence of avascular necrosis is closely related to the time hip remains dislocated, and it is reduced seriously if reduction is carried out within 6 hours from 50% to 0–5% [3, 4]. In follow-up, magnetic resonance imaging (MRI) should be used to exclude avascular necrosis. The patients after traumatic hip dislocation are at high risk for the development of early posttraumatic osteoarthritis and should be followed up for a long term [4, 6].

In isolated traumatic hip dislocation, traction is emphasized to be unnecessary. Full-weight bearing initiating time to the hip has been a subject of debate. It was stated that late full-weight bearing application to the hip joint did not change the risk of avascular necrosis and prognosis [7]. Recently few studies demonstrated that early mobilization gives better results [6, 7]. In our case, no traction was applied for the patient and early passive hip exercises were directly initiated after the reduction.

We concluded that immediate reduction of hip joints and early passive exercises followed with active-assisted range of motion were very useful for a successful treatment and for an early full-weight bearing mobilization.
